# RETRACTED: Edwards et al. The Lysine Methyltransferase SMYD2 Is Required for Definite Hematopoietic Stem Cell Production in the Mouse Embryo. *Vet. Sci.* 2020, *7*, 100

**DOI:** 10.3390/vetsci13090966

**Published:** 2026-09-15

**Authors:** Melissa A. Edwards, Mark A. Brown, Ilham Alshiraihi, Dillon K. Jarrell, Haley O. Tucker

**Affiliations:** 1 Cell and Molecular Biology Program, Colorado State University, Fort Collins, CO 80523, USA; melissa.edwards@colostate.edu (M.A.E.); mark.brown@colostate.edu (M.A.B.); alshiraihi@gmail.com (I.A.); 2 Department of Molecular Biosciences, The University of Texas at Austin, 1 University Station A5000, Austin, TX 78712, USA; 3 Department of Clinical Sciences, Colorado State University, Fort Collins, CO 80523, USA; 4 Department of Bioengineering, University of Colorado Anschutz Medical Campus, Aurora, CO 80045, USA; dillon.jarrell@cuanschutz.edu

The journal retracts the article titled “The Lysine Methyltransferase SMYD2 Is Required for Definite Hematopoietic Stem Cell Production in the Mouse Embryo” [1], cited above.

Following the publication, concerns were brought to the attention of the Editorial Office about the reliability of the data presented in this article [1].

In accordance with the journal’s standard procedure, the Editorial Office and Editorial Board conducted an investigation that identified an apparent similarity between flow cytometry panels presented in Figure 1F of this article and panels published previously in an article by a different authorship group. The investigation also identified additional irregularities within the flow cytometry data. The authors cooperated with the investigation and acknowledged concerns regarding the affected figure; however, they were unable to provide the original raw data for independent verification.

Given the unresolved concerns regarding the originality and reliability of this data, together with the absence of raw data to support their verification, the Editorial Board has lost confidence in the reliability of the findings and has decided to retract this publication, as per MDPI’s retraction policy.

This retraction was approved by the Editor-in-Chief of the *Veterinary Sciences* journal.

The authors have been informed of this retraction and have indicated their disagreement with this decision.
